# Comprehensive analysis of interleukin-8 gene polymorphisms and periodontitis susceptibility

**DOI:** 10.18632/oncotarget.16922

**Published:** 2017-04-07

**Authors:** Xiao-Bing Ni, Cheng Jia, He-Dong Yu, Yan-Qin Li, Xian-Tao Zeng, Wei-Dong Leng

**Affiliations:** ^1^ Department of Stomatology, Taihe Hospital, Hubei University of Medicine, Shiyan 442000, China; ^2^ Department of Orthodontics, The Third Hospital of Hebei Medical University, Shijiazhuang 050051, China

**Keywords:** interleukin-8, periodontitis, periodontal diseases, meta-analysis, polymorphism

## Abstract

**Background:**

Associations between interleukin-8 (IL-8) gene polymorphisms and periodontitis susceptibility have been investigated in many published studies, but the conclusions are still inconsistent. Therefore, we performed this systematic review and meta-analysis to review which polymorphisms have been researched and to obtain a precise result of the same polymorphism from different studies.

**Results:**

Finally 10 publications involving 1938 patients and 1569 controls were yielded, including 12 polymorphisms. Six studies investigated rs4073 polymorphism; two focused on rs2227306 and rs2227307; two referred to rs2227532 and T-738A; one detected rs2230054, rs1126579 and rs1126580; one inspected A2767T, T_1_1722T_2_ and C1633T, and one for rs2234671 polymorphism. Of them, IL-8 C1633T and rs1126580 polymorphisms showed positive association while the other ten polymorphisms revealed negative results.

**Materials and methods:**

A comprehensive literature search from PubMed, Web of Science, and Chinese National Knowledge Infrastructure was conducted for all potentially relevant studies published before January 2, 2017. Two authors selected the studies and extracted data. The pooled analysis was conducted using the RevMan 5.1 software if a polymorphism was reported by two or more studies.

**Conclusions:**

Based on current evidence, the IL-8 rs4073, A2767T, T_1_1722T_2_, rs2234671, rs2230054, rs1126579, rs2227306, rs2227307, rs2227532, and T-738A polymorphisms were not associated with periodontitis susceptibility; the IL-8 C1633T and rs1126580 polymorphisms were associated with increased risk of periodontitis.

## INTRODUCTION

Interleukin (IL) plays an important role in mediating immune and inflammatory responses, and periodontitis is known as a chronic infectious disease. Hence, the genes and their variants (polymorphisms) of IL may be the important determinants of pathogenesis of periodontitis [1]. There are a large number of publications reporting the association between IL gene polymorphisms and periodontitis, some related meta-analyses have been performed for IL-1 polymorphisms [2–6], IL-4 polymorphisms [7], IL-6 polymorphisms [8], IL-10 polymorphisms [9–10], and IL-18 polymorphisms [11]. IL-8 gene is a component of IL genes with more than ten polymorphisms [12]. Numerous studies exploring the association between IL-8 gene polymorphisms and periodontitis have been published, but the results of previous studies of same polymorphism were inconsistent. Yang et al. [13] in 2016 performed a meta-analysis to investigate the effect of rs4073 (A251T/T-353A) on periodontitis susceptibility and found a positive association. The meta-analysis by Chen et al. [14] in 2015 focused on the rs4073 (A251T/T-353A) and rs2227532 (T-845C) polymorphisms which also revealed a positive association. Obviously, many other polymorphisms of IL-8 gene has not been explored, so we performed this analysis for further assessing the relationship between all IL-8 gene polymorphisms and periodontitis; additionally, we also reviewed which polymorphisms have been investigated. The present study followed the Preferred Reporting Items for Systematic Reviews and Meta-Analyses (PRISMA) statement [15–16].

## RESULTS

### Study characteristics

**Figure 1:**
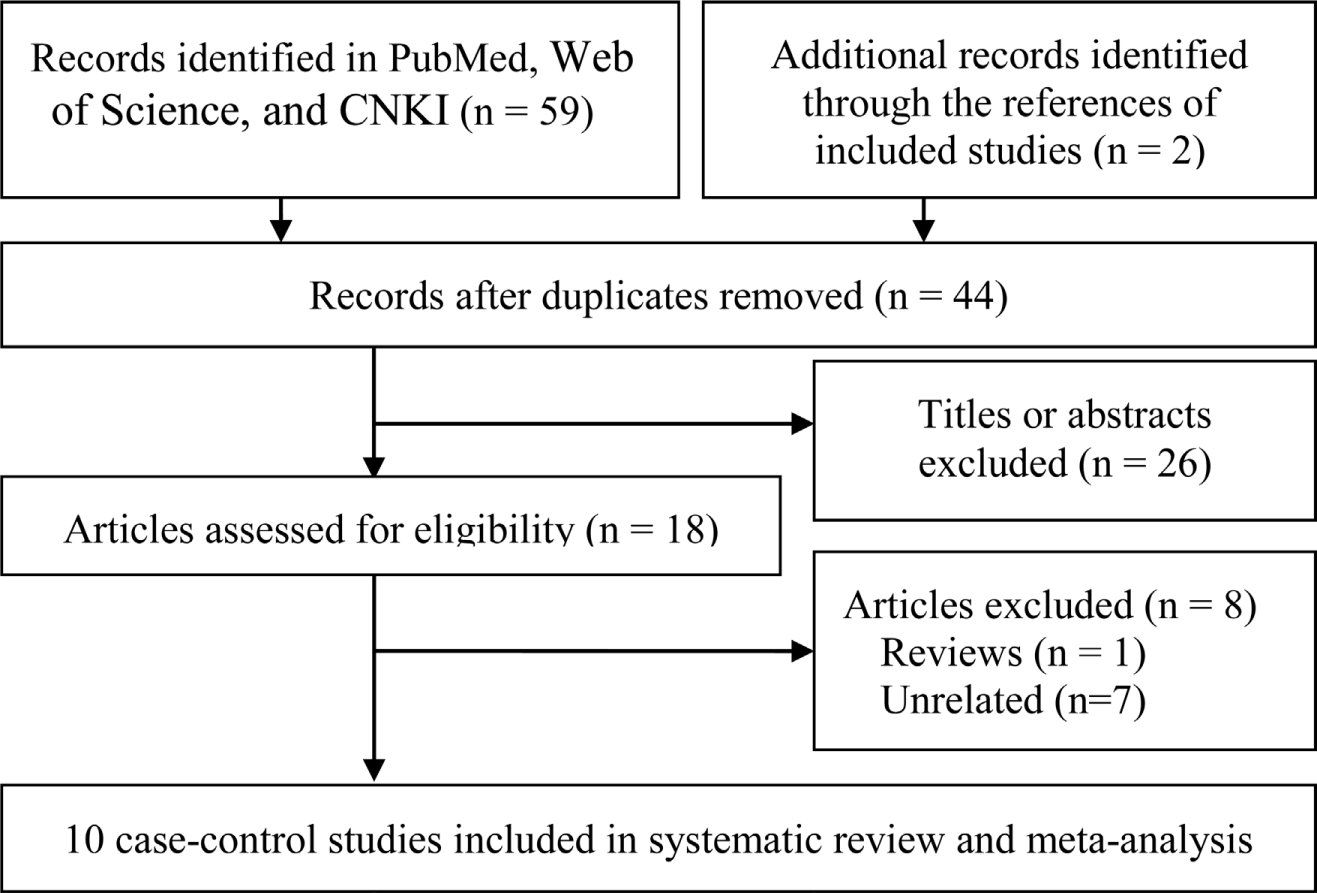
Study selection flow chart

The initial search identified 61 publications and finally 10 articles [17–26] were included in the systematic review and meta-analysis. The literature retrieval and selection are shown in Figure 1.

The characteristics and relevant data of the included studies are shown in Table 1 and Table 2. All studies were case-control studies involving 12 polymorphisms: rs4073 (A251T/T-353A), A2767T, T_1_1722T_2_, C1633T, rs2234671, rs2230054 (C785T), rs1126579 (T1208C), rs1126580 (G1440A), rs2227306 (C781T), rs2227307 (G396T), rs2227532 (T-845C), and T-738A. Eight studies focused on chronic periodontitis (CP) [17–21, 24–26]; contained CP and agreessive periodontitis (AgP) [22], and one only referred to AgP [23]. There were a total of 1938 patients and 1569 controls.

**Table 1:** Characteristics of included studies

Reference	Country (ethnicity)	Type	Polymorphism	Smoking status	Source of control	Genotyping method	HWE
Kim 2009	Brazil (Brazilian)	CP	rs4073 (A251T/T-353A)	Mixed	Healthy	PCR-SSP	Yes
Kim 2010	Brazil (Brazilian)	CP	rs2227532 (T-845C), T-738A	Mixed	Healthy	PCR–RFLP	Yes
Viana 2010	Brazil (Brazilian)	CP	rs2230054 (C785T), rs1126579 (T1208C), rs1126580 (G1440A)	Mixed	Healthy	PCR-SSP	Yes
Andia 2011	Brazil (Brazilian)	CP	rs4073 (A251T)	No	Healthy	PCR	Yes
Scarel-Caminaga 2011	Brazil (Brazilian)	CP	rs2227306 (C781T), rs2227307 (G396T)	Mixed	Healthy	PCR–RFLP	Yes
Scarel-Caminaga 2011	Brazil (Brazilian)	CP	rs2234671	Mixed	Healthy	PCR-SSP	Yes
Houshmand 2012	Iran (Caucasian)	CP and AgP	rs4073 (A251T), A2767T, C781T, T11722T2, G396T, C1633T	NA	Healthy	PCR	Yes
Andia 2013	Brazil (Brazilian)	AgP	rs4073 (A251T)	No	Healthy	PCR	Yes
Khosropanah 2013	Iran (Caucasian)	CP	rs4073 (A251T)	No	Healthy	PCR	Yes
Sippert 2013	Brazil (Brazilian)	CP	rs4073 (T-353A), rs2227532 (T-845C), T-738A	Mixed	Healthy	PCR–RFLP	Yes

AgP, aggressive periodontitis; CP, chronic periodontitis; HWE, Hardy Weinberg equilibrium; Mixed, included smokers and non-smokers; NA, not available; PCR-SSP, polymerase chain reaction- single strand polymorphism; PCR-RFLP, PCR- restriction fragment length polymorphism.

**Table 2:** The data of included studies

Polymorphism	Type	Case/Control			
**rs4073 (A251T/T-353A)**					
		AA	TA	TT	N
Andia 2011	CP	21/13	135/57	25/38	181/108
Andia 2013	AgP	11/13	50/57	15/38	76/108
Houshmand 2012	CP and AgP	40/10	55/120	12/69	107/199
Sippert 2013	CP	34/53	62/92	28/42	124/187
Khosropanah 2013	CP	41/12	101/17	85/11	227/40
Kim 2009	CP	56/36	146/120	66/64	268/220
**A2767T**					
		AA	AT	TT	N
Houshmand 2012	CP and AgP	8/20	59/110	40/69	107/199
**rs2227306 (C781T)**					
		CC	CT	TT	N
Houshmand 2012	CP and AgP	42/70	63/129	2/0	107/199
Scarel-Caminaga 2011	CP	141/105	112/96	17/22	270/223
**T11722T2**					
		T1T1	T1T2	T2T2	N
Houshmand 2012	CP and AgP	103/199	4/0	0/0	107/199
**rs2227307 (G396T)**					
		GG	GT	TT	N
Houshmand 2012	CP and AgP	28/10	55/120	24/69	107/199
Scarel-Caminaga 2011	CP	36/29	120/125	114/69	270/223
**C1633T**					
		CC	CT	TT	N
Houshmand 2012	CP and AgP	22/90	21/0	64/109	107/199
**rs2234671**					
		GG	GC	CC	N
Scarel-Caminaga 2011	CP	161/164	31/28	3/3	195/195
**rs2227532 (T-845C)**					
		TT	TC	CC	N
Sippert 2013	CP	117/183	6/4	1/0	124/187
Kim 2010	CP	137/127	80/55	1/0	218/182
**T-738A**					
		TT	TA	AA	N
Sippert 2013	CP	123/186	1/1	0/0	124/187
Kim 2010		61/59	155/122	2/1	218/182
**rs2230054 (C785T)**					
		CC	CT	TT	N
Viana 2010	CP	20/17	244/193	8/5	272/215
**rs1126579 (T1208C)**					
		TT	TC	CC	N
Viana 2010	CP	31/21	198/170	43/24	272/215
**rs1126580 (G1440A)**					
		GG	GA	AA	N
Viana 2010	CP	13/32	194/119	65/64	272/215

AgP, aggressive periodontitis; CP, chronic periodontitis; N, sample size.

### rs4073 polymorphism

**Figure 2:**
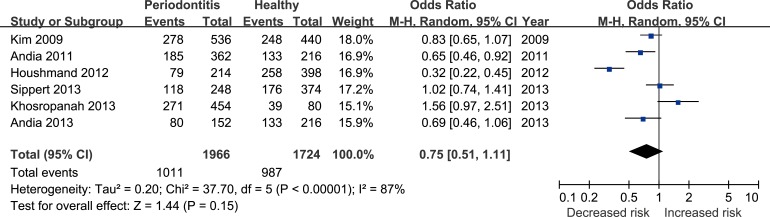
Forest plot for the T vs. A genetic model in IL-8 rs4073 polymorphism

**Figure 3:**
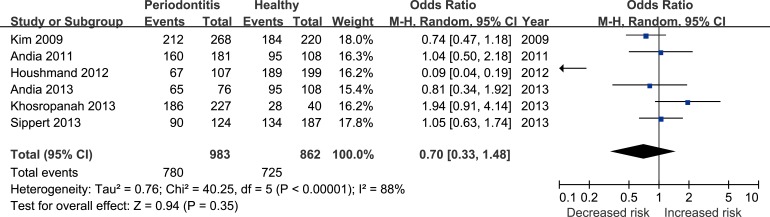
Forest plot for the TA+TT vs. AA genetic model in IL-8 rs4073 polymorphism

**Figure 4:**
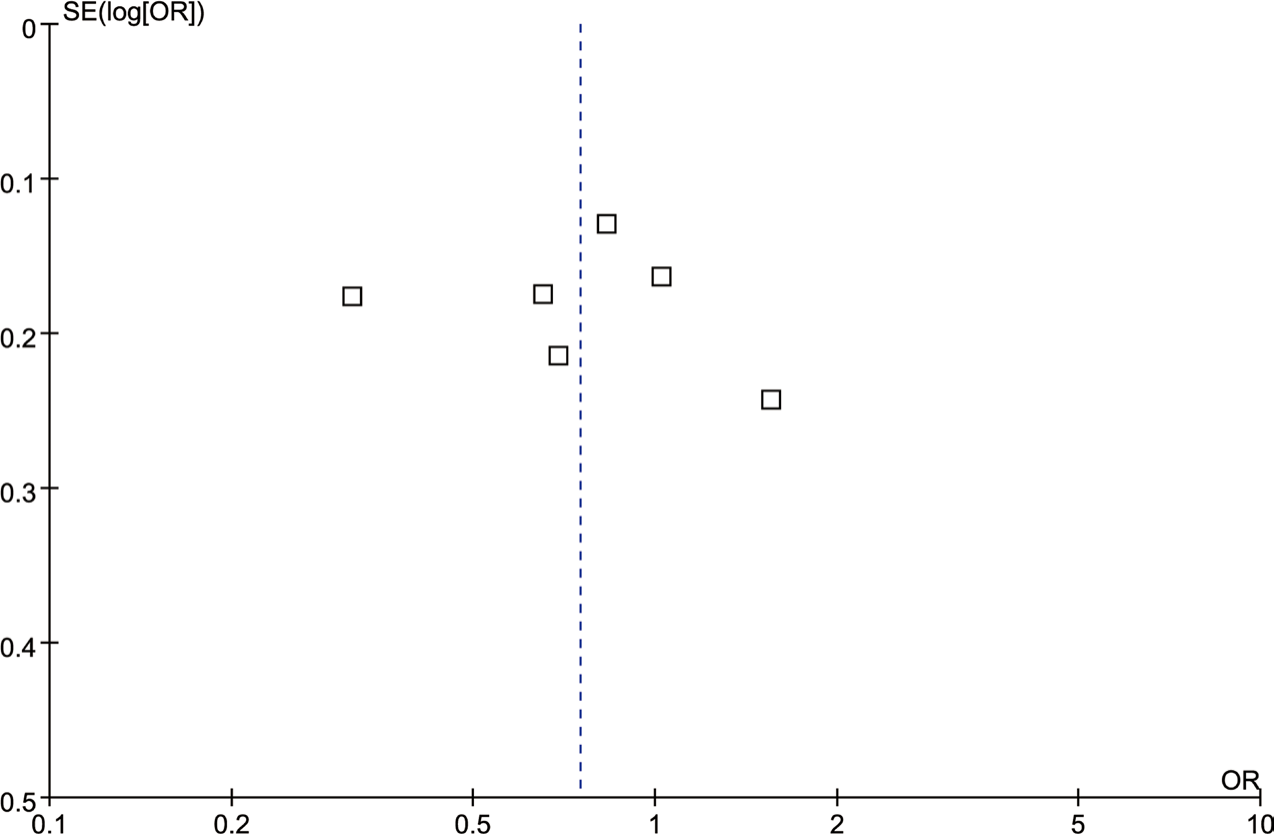
Funnel plot for the T vs. A genetic model in IL-8 rs4073 polymorphism

Six studies [17, 22, 24–27] reported the correlation between rs4073 (A251T/T-353A) polymorphism and periodontitis. The meta-analysis based on random effect model indicated no significant association between them [T vs. A: odds ratio (OR) = 0.75, 95% confidence interval (CI) = 0.51–1.11, Figure 2; TA+TT vs. AA: OR = 0.70, 95%CI = 0.33-1.48, Figure 3)]. The funnel plot was symmetrical (Figure 4).

### A2767T polymorphism

One study [22] on A2767T polymorphism reported that it was not associated with periodontitis risk (T vs. A: OR = 1.12, 95%CI = 0.79–1.580, *p* = 0.52; TA+TT vs. AA: OR = 1.38, 95% CI = 0.59–3.25, *p* = 0.46).

### rs2227306 polymorphism

Meta-analysis of two studies [21–22] showed there was no significant association between rs2227306 (C781T) polymorphism and periodontitis risk. The specific results were presented in Table 3.

**Table 3:** Results of meta-analysis of IL-8 gene rs4037, rs2227306, rs2227307, rs2227532 and T-738A polymorphisms

Polymorphism	Genetic model	No.	Heterogeneity		Effect model	Meta-analysis	
			*I*^2^(%)	*p*^h^		OR (95% CI)	*p*
rs4073 (A251T/T-353A)	T vs. A	6	87	< 0.01	Random	0.75 (0.51–1.11)	0.15
	TA + TT vs. AA	6	88	< 0.01	Random	0.70 (0.33–1.48)	0.35
rs2227306 (C781T)	T vs. C	2	97	< 0.01	Random	0.44 (0.10–1.99)	0.29
	TC + TT vs. CC	2	0	0.92	Fixed	0.82 (0.62–1.10)	0.18
rs2227307 (G396T)	T vs. G	2	94	< 0.01	Random	0.90 (0.73–1.10)	0.31
	TG + TT vs. GG	2	94	< 0.01	Random	0.39 (0.06–2.44)	0.31
rs2227532 (T-845C)	C vs. T	2	44	0.18	Fixed	1.41 (0.99–2.01)	0.06
	CT+CC vs. TT	2	6	0.3	Fixed	1.47 (0.99–2.18)	0.06
T-738A	A vs. T	2	0	0.83	Fixed	1.11 (0.83–1.49)	0.46
	AT + AA vs. TT	2	0	0.89	Fixed	1.24 (0.81–1.89)	0.32

No., number of studies; CI, confidence interval; OR, odds ratio.

### T_1_1722T_2_ polymorphism

One study [22] detecting the relationship between the T_1_1722T_2_ polymorphism and periodontitis susceptibility suggested that there was no significant association between them (T_2_ vs. T_1_: OR = 17.04, 95%CI = 0.91–317.97, *p* = 0.06; T_2_T_1_+T_2_T_2_ vs. T_1_T_1_: OR = 17.35, 95%CI = 0.93-325.33, *p* = 0.06).

### rs2227307 polymorphism

Table 3 showed the results of rs2227307 (G396T) polymorphism and periodontitis risk, and the meta-analysis of two studies [21–22] indicated non-significant association.

### C1633T polymorphism

The results from one study [22] on C1633T polymorphism indicated that this was an increased risk of periodontitis (T vs. C: OR = 1.89, 95%CI = 1.33–2.69, *p* < 0.01; TC+TT vs. CC: OR = 3.19, 95%CI = 1.85–5.51, *p* < 0.01).

### rs2234671 polymorphism

One study [20] involving rs2234671 polymorphism showed it was not related to periodontitis risk (C vs. G: OR = 1.10, 95%CI = 0.67–1.79, *p* = 0.71; CG+CC vs. GG: OR = 1.12, 95%CI = 0.66–1.90, *p* = 0.68).

### rs2227532 polymorphism

Two studies [18, 25] reported the data for rs2227532 (T-845C) polymorphism, and the meta-analysis displayed this polymorphism was had no significant impact on periodontitis (Table 3).

### T-738A polymorphism

The T-738A polymorphism was evaluated by two studies [18, 25] and the pooled results uncovered there was non-significant association of T-738A polymorphism with periodontitis (Table 3).

### rs2230054 polymorphism

The rs2230054 (C785T) polymorphism was assessed in one study [19] which revealed non-significant association between this polymorphism and periodontitis (T vs. C: OR = 1.02, 95%CI = 0.79–1.32, *p* = 0.86; TC+TT vs. CC: OR = 1.08, 95%CI = 0.55–2.12, *p* = 0.82).

### rs1126579 polymorphism

The rs1126579 (T1208C) polymorphism was reported by one study [19]. The results indicated that this polymorphism was not implicated in periodontitis (C vs. T: OR = 1.06, 95%CI = 0.82–1.37, *p* = 0.64; CT+CC vs. TT: OR = 0.84, 95%CI = 0.47–1.51, *p* = 0.56).

### rs1126580 polymorphism

The results from one study [19] demonstrated that rs1126580 (G1440A) polymorphism might be associated with the increased risk of periodontitis (AG+AA vs. GG: OR = 3.48, 95%CI = 1.78–6.82, *p* < 0.01).

## DISCUSSION

IL-8 gene located on chromosome 4q12-21 contains four exons and three introns which possesses many functional polymorphisms [12, 28]. Published meta-analyses indicate that IL-8 gene polymorphisms are associated with some diseases, such as gastric cancer [29], oral cancer [30], and peptic ulcer disease [31]. The first study assessing IL-8 rs4037 polymorphism and periodontitis was published by Kim et al. [17] in 2011. Our systematic review and meta-analysis included 10 publications involving 3507 individuals, investigating the correlations between 12 polymorphisms of IL-8 gene and periodontitis. The results showed that the IL-8 rs4073 (A251T/T-353A), A2767T, T_1_1722T_2_, rs2234671, rs2230054 (C785T), rs1126579 (T1208C), rs2227306 (C781T), rs2227307 (G396T), rs2227532 (T-845C), and T-738A polymorphisms were not significantly related to periodontitis susceptibility; however, there was a significant difference in the IL-8 C1633T and rs1126580 (G1440A) polymorphisms between the periodontitis patients and healthy control groups.

Compared with published two meta-analyses [13–14] on this topic, the major strength of our study is that it is the first comprehensive meta-analysis on IL-8 gene polymorphisms and periodontitis risk. We believe it gives a useful summary of current data regarding the relationship between IL-8 gene polymorphisms and periodontitis risk, and provides improved clinical clarity to obtain a solid evidence base for the diagnosis and treatment of periodontitis. As to the included studies, sample size for each polymorphism was very small, so the relevant researches are suggested to be conducted in the future. Since there are more than 15 polymorphisms of IL-8 gene have been characterized [29], the association between additional polymorphisms and risk of periodontitis should be investigated. The AgP and CP are two different types of periodontitis and the former type is considered as a genetically inherited disease [2, 32]. Hence, the further studies should report the data for CP and AgP separately.

The major limitation of our study was the numbers of included studies and sample size. Of these 12 polymorphisms, only IL-8 rs4073 polymorphism was involved in 6 studies while the others referred to one or two studies. Correspondingly, the sample size for each polymorphism was relatively small, which restrained the confidence of current results. Moreover, the subgroup analyses based on difference between ethnicity, smoking status, type of periodontitis, or other factors couldn't be conducted to investigate the source of heterogeneity, which might bias the results. Because of the limited numbers of included studies, we only assessed the publication bias of those investigating the IL-8 rs4073 polymorphism. Secondly, systematic review and meta-analysis is an observational study which was restricted by the quality of primary studies [6, 16, 33–35]. Although we had conducted a more comprehensive search, our study could not escape from this limitation. Lastly, our results were based on unadjusted data and the original data were sufficient, hence the evaluation of the effects of the gene - gene or gene - environment interactions was neglected. These limitations mentioned above might affect our final conclusions.

In conclusion, our meta-analysis suggests that the IL-8 rs4073, A2767T, T_1_1722T_2_, rs2234671, rs2230054, rs1126579, rs2227306, rs2227307, rs2227532, and T-738A polymorphisms are not associated with periodontitis while the IL-8 C1633T and rs1126580 polymorphisms may elevate the susceptibility of periodontitis based on the currently available evidences. However, for considering that the studies included in our meta-analysis were based on small numbers, the current results should be treated with caution, and the results may change with the larger sample size in future. Due to these limitations, more well designed, studies with larger sample size, and adjusted risk factors are required to further validate our results.

## MATERIALS AND METHODS

### Eligible criteria

We included the studies which met all of the following criteria: (1) the patients were clearly diagnosed as periodontitis (CP and/or AgP) and the controls were periodontitis-free or periodontal healthy; (2) at least one of the IL-8 polymorphisms and periodontitis susceptibility were evaluated, using a case-control or cohort study design; (3) the studies reported full data for necessary genotypes in each group or contained sufficient data to calculate them. If the same institute published two or more publications, we treated them as independent ones and chose the more comprehensive one.

### Search strategy

A comprehensive literature search was performed in PubMed, Web of Science, and Chinese National Knowledge Infrastructure (CNKI) up to January 2, 2017. The following key words were used: IL-8, interleukin-8, interleukin 8, periodontal disease, periodontitis, variant, and polymorphism. Moreover, all listed references of included studies and recently reviews were retrieved for any additional relevant studies. No language restriction was applied in the search process.

### Data extraction

Study selection and data extraction were performed by two authors and any discrepancy was resolved by discussion. The following data were extracted from each included study: surname of first author, year of publication, study design, country and ethnicity of study population, demographics, periodontitis type, smoking status, number of cases and controls, polymorphism, genotype distribution, source of controls, genotyping method, and Hardy-Weinberg equilibrium (HWE) for controls.

### Data analysis

The OR and its 95% CI were calculated to estimate the relationship between the IL-8 polymorphisms and periodontitis risk. All studies used a allele genetic model and a dominant genetic model [5]. If a polymorphism was reported in two or more studies, a meta-analysis was conducted. Heterogeneity was assessed using the Cochran's *Q* statistic and *I*^2^ statistic [15, 36], with values of *P* ≥ 0.1 and *I*^2^ < 50% indicating acceptable heterogeneity. If no significant heterogeneity existed, the fixed effect model was used; otherwise, the random-effects model was used. If the number of included studies was available, we conducted subgroup analyses on the ethnicity, smoking status, and periodontitis type. Publication bias was assessed by funnel plot [37]. All analyses were performed using RevMan 5.1 software [38–39].

## References

[1] Laine ML, Crielaard W, Loos BG (2012). Genetic susceptibility to periodontitis. Periodontol 2000.

[2] Wang WF, Shi J, Chen SJ, Niu YM, Zeng XT (2014). Interleukin-1alpha −899 (4845) C—>T polymorphism is not associated with aggressive periodontitis susceptibility: A meta-analysis based on 19 case-control studies. Biomed Rep.

[3] Mao M, Zeng XT, Ma T, He W, Zhang C, Zhou J (2013). Interleukin-1alpha −899 (4845) C—>T polymorphism increases the risk of chronic periodontitis: evidence from a meta-analysis of 23 case-control studies. Gene.

[4] Deng JS, Qin P, Li XX, Du YH (2013). Association between interleukin-1beta C (3953/4)T polymorphism and chronic periodontitis: evidence from a meta-analysis. Hum Immunol.

[5] Karimbux NY, Saraiya VM, Elangovan S, Allareddy V, Kinnunen T, Kornman KS, Duff GW (2012). Interleukin-1 gene polymorphisms and chronic periodontitis in adult whites: a systematic review and meta-analysis. J Periodontol.

[6] Huang W, He BY, Shao J, Jia XW, Yuan YD (2017). Interleukin-1β rs1143627 polymorphism with susceptibility to periodontal disease. Oncotarget.

[7] Yan Y, Weng H, Shen ZH, Wu L, Zeng XT (2014). Association between interleukin-4 gene −590 c/t, −33 c/t, and 70-base-pair polymorphisms and periodontitis susceptibility: a meta-analysis. J Periodontol.

[8] Shao MY, Huang P, Cheng R, Hu T (2009). Interleukin-6 polymorphisms modify the risk of periodontitis: a systematic review and meta-analysis. J Zhejiang Univ Sci B.

[9] Albuquerque CM, Cortinhas AJ, Morinha FJ, Leitão JC, Viegas CA, Bastos EM (2012). Association of the IL-10 polymorphisms and periodontitis: a meta-analysis. Mol Biol Rep.

[10] Zhong Q, Ding C, Wang M, Sun Y, Xu Y (2012). Interleukin-10 gene polymorphisms and chronic/aggressive periodontitis susceptibility: a meta-analysis based on 14 case-control studies. Cytokine.

[11] Li ZG, Li JJ, Sun CA, Jin Y, Wu WW (2014). Interleukin-18 promoter polymorphisms and plasma levels are associated with increased risk of periodontitis: a meta-analysis. Inflamm Res.

[12] Bickel M (1993). The role of interleukin-8 in inflammation and mechanisms of regulation. J Periodontol.

[13] Yang ZJ, Tang XP, Lai QG, Ci JB, Yuan KF (2016). Interleukin-8 −251A/T polymorphism and periodontitis susceptibility: a meta-analysis. Genet Mol Res.

[14] Chen X, Huang J, Zhong L, Ding C (2015). Quantitative assessment of the associations between interleukin-8 polymorphisms and periodontitis susceptibility. J Periodontol.

[15] Moher D, Liberati A, Tetzlaff J, Altman DG, Group P (2009). Preferred reporting items for systematic reviews and meta-analyses: the PRISMA statement. BMJ.

[16] Zhai Y, Dai Z, He H, Gao F, Yang L, Dong Y, Lu J (2016). A PRISMA-compliant meta-analysis of MDM4 genetic variants and cancer susceptibility. Oncotarget.

[17] Kim YJ, Viana AC, Curtis KM, Orrico SR, Cirelli JA, Scarel-Caminaga RM (2009). Lack of association of a functional polymorphism in the interleukin 8 gene with susceptibility to periodontitis. DNA Cell Biol.

[18] Kim YJ, Viana AC, Curtis KM, Orrico SR, Cirelli JA, Mendes-Junior CT, Scarel-Caminaga RM (2010). Association of haplotypes in the IL8 gene with susceptibility to chronic periodontitis in a Brazilian population. Clin Chim Acta.

[19] Viana AC, Kim YJ, Curtis KM, Renzi R, Orrico SR, Cirelli JA, Scarel-Caminaga RM (2010). Association of haplotypes in the CXCR2 gene with periodontitis in a Brazilian population. DNA Cell Biol.

[20] Scarel-Caminaga RM, Curtis KM, Renzi R, Sogumo PM, Anovazzi G, Viana AC, Kim YJ, Orrico SR, Cirelli JA (2011). Variation in the CXCR1 gene (IL8RA) is not associated with susceptibility to chronic periodontitis. J Negat Results Biomed.

[21] Scarel-Caminaga RM, Kim YJ, Viana AC, Curtis KM, Corbi SC, Sogumo PM, Orrico SR, Cirelli JA (2011). Haplotypes in the interleukin 8 gene and their association with chronic periodontitis susceptibility. Biochem Genet.

[22] Houshmand B, Hajilooi M, Rafiei A, Bidgoli M, Soheilifar S (2012). Evaluation of IL-8 gene polymorphisms in patients with periodontitis in Hamedan, Iran. Dent Res J (Isfahan).

[23] Andia DC, Letra A, Casarin RC, Casati MZ, Line SR, de Souza AP (2013). Genetic analysis of the IL8 gene polymorphism (rs4073) in generalized aggressive periodontitis. Arch Oral Biol.

[24] Khosropanah H, Sarvestani EK, Mahmoodi A, Golshah M (2013). Association of IL-8 (−251 a/t) gene polymorphism with clinical parameters and chronic periodontitis. J Dent (Tehran).

[25] Sippert EA, de Oliveira e Silva C, Visentainer JE, Sell AM (2013). Association of duffy blood group gene polymorphisms with IL8 gene in chronic periodontitis. PLoS One.

[26] Andia DC, de Oliveira NF, Letra AM, Nociti FH, Line SR, de Souza AP (2011). Interleukin-8 gene promoter polymorphism (rs4073) may contribute to chronic periodontitis. J Periodontol.

[27] Kalischuk LD, Inglis GD (2011). Comparative genotypic and pathogenic examination of Campylobacter concisus isolates from diarrheic and non-diarrheic humans. BMC Microbiol.

[28] Hull J, Rowlands K, Lockhart E, Sharland M, Moore C, Hanchard N, Kwiatkowski DP (2004). Haplotype mapping of the bronchiolitis susceptibility locus near IL8. Hum Genet.

[29] Cheng D, Hao Y, Zhou W, Ma Y (2013). Positive association between Interleukin-8 −251A > T polymorphism and susceptibility to gastric carcinogenesis: a meta-analysis. Cancer Cell Int.

[30] Wang Z, Wang C, Zhao Z, Liu F, Guan X, Lin X, Zhang L (2013). Association between −251A>T polymorphism in the interleukin-8 gene and oral cancer risk: a meta-analysis. Gene.

[31] Yin YW, Hu AM, Sun QQ, Zhang BB, Wang Q, Liu HL, Zeng YH, Xu RJ, Zhang SJ, Shi LB (2013). Association between interleukin-8 gene −251 T/A polymorphism and the risk of peptic ulcer disease: a meta-analysis. Hum Immunol.

[32] Hart TC, Pallos D, Bozzo L, Almeida OP, Marazita ML, O'Connell JR, Cortelli JR (2000). Evidence of genetic heterogeneity for hereditary gingival fibromatosis. J Dent Res.

[33] Zeng X, Zhang Y, Kwong JS, Zhang C, Li S, Sun F, Niu Y, Du L (2015). The methodological quality assessment tools for preclinical and clinical studies, systematic review and meta-analysis, and clinical practice guideline: a systematic review. J Evid Based Med.

[34] Zeng XT, Liu DY, Kwong JS, Leng WD, Xia LY, Mao M (2015). Meta-Analysis of Association Between Interleukin-1β C-511T Polymorphism and Chronic Periodontitis Susceptibility. J Periodontol.

[35] Zeng XT, Leng WD, Lam YY, Yan BP, Wei XM, Weng H, Kwong JS (2016). Periodontal disease and carotid atherosclerosis: A meta-analysis of 17,330 participants. Int J Cardiol.

[36] Higgins JP, Thompson SG, Deeks JJ, Altman DG (2003). Measuring inconsistency in meta-analyses. BMJ.

[37] Egger M, Davey Smith G, Schneider M, Minder C (1997). Bias in meta-analysis detected by a simple, graphical test. BMJ.

[38] Leng WD, Wen XJ, Kwong JS, Huang W, Chen JG, Zeng XT (2016). COX-2 rs689466, rs5275, and rs20417 polymorphisms and risk of head and neck squamous cell carcinoma: a meta-analysis of adjusted and unadjusted data. BMC Cancer.

[39] Zeng XT, Xia LY, Zhang YG, Li S, Leng WD, Kwong JS (2016). Periodontal Disease and Incident Lung Cancer Risk: A Meta-Analysis of Cohort Studies. J Periodontol.

